# Supplementary figures and images for: Attenuation correction using 3D deep convolutional neural network for brain 18F-FDG PET/MR: Comparison with Atlas, ZTE and CT based attenuation correction
Source: PLoS One. 2019 Oct 7;14(10):e0223141. doi: 10.1371/journal.pone.0223141 (PMC6779234; doi:10.1371/journal.pone.0223141)

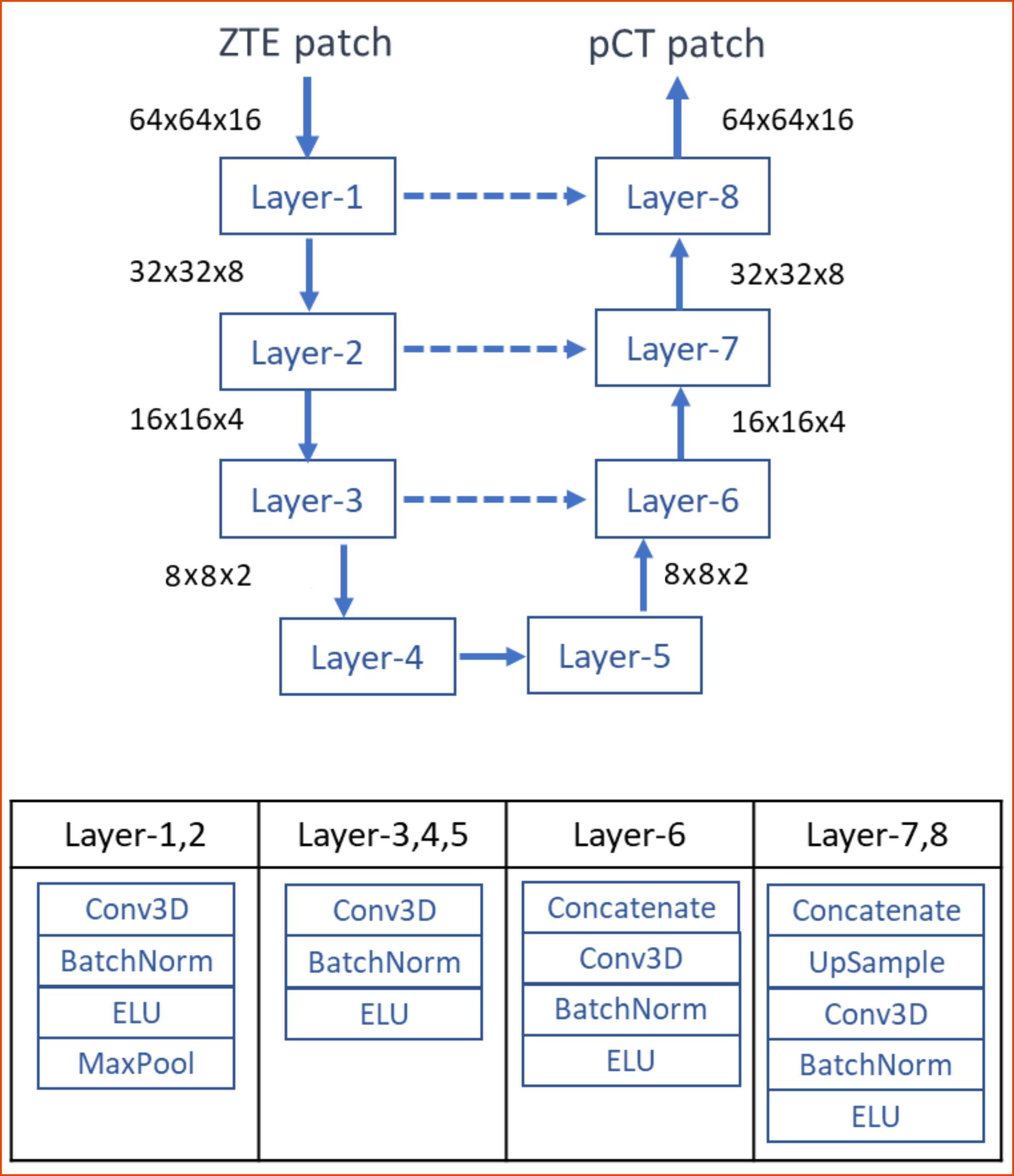

Supplement: S1 Fig — It takes as input a 64x64x16 patch from ZTE that goes through an encoding followed by a decoding path which concatenated by concatenations, and produces a 64x64x16 pseudoCT. (TIFF) [file pone.0223141.s001.tiff]

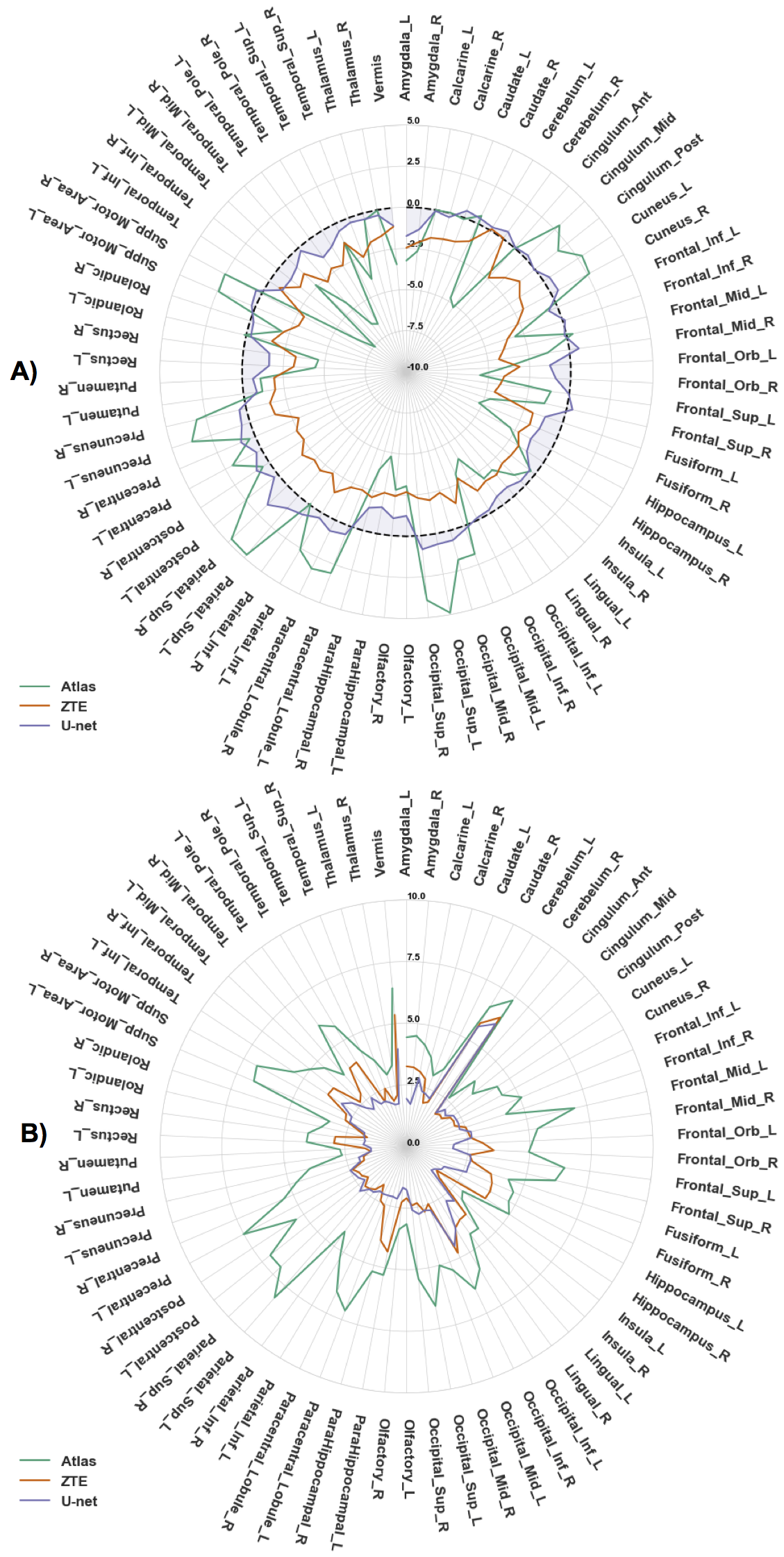

Supplement: S2 Fig — Radar plot among patients (n = 47) for Atlas, ZTE and U-net based attenuation correction within the 70 VOIs from AAL template. (TIFF) [file pone.0223141.s002.tiff]
